# Preoperative Echocardiographic Assessment in Elective Surgery Patients: A Cross-Sectional Study From Duhok, Iraqi Kurdistan

**DOI:** 10.7759/cureus.70395

**Published:** 2024-09-28

**Authors:** Ameen M Mohammad, Hakar M Mohammed

**Affiliations:** 1 Department of Internal Medicine, University of Duhok, Duhok, IRQ; 2 Department of Cardiology, Azadi Teaching Hospital, Duhok, IRQ

**Keywords:** cardiac abnormalities, echocardiography, iraq, kurdistan region, preoperative patients, surgery

## Abstract

Background and aim

Echocardiography plays a pivotal role in the preoperative risk assessment of patients undergoing various surgical procedures. Therefore, we conducted this study to detect various cardiac abnormalities through a preoperative echocardiographic study for cases undergoing elective surgeries in Duhok, Iraqi Kurdistan.

Methods

This cross-sectional study assessed echocardiographic findings in preoperative patients at Azadi Teaching Hospital, Duhok, Kurdistan Region of Iraq, from 2023 to 2024. The study encompasses 468 adult patients. We gathered clinicodemographic characteristics, indications of referral to a preoperative echo study, and the echocardiographic findings, particularly regional wall motion abnormalities, valvular heart diseases, and heart failure (systolic or diastolic/left ventricular hypertrophy).

Results

In a cohort of 468 patients, 205 (43.80%) were aged 35-54 years, and 269 (57.48%) were female. Most patients (366; 78.21%) resided in Duhok. Nearly only half of the cases 219 (46.79%) had clear preoperative echostudy indications. A total of 289 (61.74%) had no remarkable echocardiographic findings. Diastolic heart failure was most prevalent at 65 (13.89%). Older cases had more prevalent echo findings in terms of valve dysfunctions and heart failure. Notable associations were found between echocardiographic abnormalities and surgical types, particularly higher regional wall motion abnormalities in genito-urinary system operations (4; 8.16%) and valvular heart disease in orthopedic surgeries (13; 15.85%).

Conclusions

Echocardiographic abnormalities were remarkably observed in patients aged 65 and older. Many cases had no clear indications for preoperative echo study and hence unremarkable echo findings. More scrutiny is indicated during referral, and focusing on older adults’ preoperative cardiac screening is recommended.

## Introduction

Several unexpected complications can occur during the perioperative period of any surgical procedure. Therefore, preoperative evaluations are crucial for predicting and preventing these complications. Cardiovascular assessment is particularly important because perioperative cardiovascular complications can sometimes be fatal [1,2].

Echocardiography plays a key role in this preoperative assessment by providing valuable information on cardiac structure, function, and hemodynamics, which aids clinicians in risk stratification, perioperative management, and surgical decision-making [3-5]. It also assesses the feasibility and safety of surgical interventions in patients with complex cardiac anatomy or significant comorbidities [6]. Key echocardiographic predictors of adverse perioperative outcomes include left ventricular ejection fraction, left ventricular hypertrophy, diastolic dysfunction, and significant valvular abnormalities [7-10]. Comparing echocardiography with other preoperative risk assessment tools like electrocardiography, stress testing, cardiac biomarkers, and computed tomography angiography can help determine its diagnostic accuracy and clinical utility [10,11].

In the region, data on echocardiographic findings in patients undergoing elective surgery is scarce. The aim of this study is to identify and characterize specific cardiac abnormalities detected through preoperative echocardiography in patients scheduled for elective surgeries in Duhok, Kurdistan Region, Iraq.

## Materials and methods

Study design and participants

This study adopts a cross-sectional design to investigate echocardiographic findings among preoperative patients undergoing surgery at Azadi Teaching Hospital, Duhok, Kurdistan Region of Iraq. A study comprised 468 patients scheduled for elective surgery between the period of October 2023 and April 2024. The study protocol was formally approved by the Ethics and Scientific Committee of the Iraqi Kurdistan Higher Council of Medical Specialization. Written informed consent was obtained from all patients upon enrollment.

Patients and methods

The study questionnaire was categorized into two sections: the first section collected data about the patient’s clinicodemographic characteristics, which included age, gender, residency, as well as past medical history of diabetes mellitus, hypertension, ischemic heart disease, and family history of ischemic heart disease. In addition, we regarded the referral to a preoperative echocardiographic study indicated based on the presence of documented prior cardiovascular conditions, abnormal preoperative electrocardiography, and the presence of suggestive cardiac symptoms or signs. The second section of the study emphasized the preoperative echocardiographic findings, which mainly focused on the presence of regional wall motion abnormalities, valvular heart disease (stenosis or regurgitation), and heart failure either diastolic or systolic. Transthoracic echocardiograms were performed using a standard protocol by experienced cardiac sonographers following the guidelines of the American Society of Echocardiography and the European Society of Echocardiography.

Inclusion and exclusion criteria

The study included patients aged 18 years or older who were scheduled for various elective surgical procedures, including cardiac, abdominal, obstetrics-gynecological, genitourinary system, and orthopedic surgeries, and who consented to participate. Exclusion criteria included emergency or urgent surgical cases, patients with difficulties in performing appropriate echocardiography, patients with implanted cardiac devices, and those with incomplete or missing questionnaire information.

Statistical analysis

Data were initially entered into Excel (Microsoft Corporation, Redmond, WA, United States) for cleaning and coding. The dataset was then transferred to GraphPad Prism (Dotmatics, Boston, MA, United States) for statistical analysis. Descriptive statistics were summarized using frequencies and percentages. The chi-square test was used to evaluate the association between echocardiographic parameters and patients’ characters, different age groups, and echocardiographic findings. A p-value of 0.05 or less was considered statistically significant.

## Results

Clinicodemographic characteristics

Table 1 provides a basic profile of the 468 patients enrolled in the study. Approximately 205 (43.80%) of the patients are aged between 35 and 54 years. Females made up 269 (57.48%) of the study cohort. A majority of the patients (366; 78.21%) reside in Duhok. Nearly half of the patients (219; 46.79%) had clear indications for echo study referral. More than half had unremarkable echocardiographic findings. A cluster of cases had cardiovascular risk factors.

**Table 1:** Clinicodemographic characteristics of the study patients (n = 468)

Variables	n	%
Age group		
20-34	16	3.42%
35-54	205	43.80%
55-64	109	23.29%
>65	138	29.49%
Gender		
Male	199	42.52%
Female	269	57.48%
Residency		
Akre	45	9.62%
Amedi	34	7.26%
Duhok	366	78.21%
Mosul	3	0.64%
Zakho	20	4.27%
Indications of referral		
Yes	219	46.79%
No	249	53.21%
Diabetes mellitus		
Yes	124	26.50%
No	344	73.50%
Hypertension		
Yes	226	48.29%
No	242	51.71%
Ischemic heart disease		
Yes	96	20.51%
No	372	79.49%
Family history of cardiovascular diseases		
Yes	43	9.19%
No	425	90.81%
Echocardiogram findings		
Abnormal echocardiograms	179	38.24%
Normal echocardiograms	289	61.76%

Echocardiographic findings

Table 2 highlights the correlation between age and echocardiographic abnormalities. The most common echocardiographic finding was diastolic heart failure (65; 13.89%), followed by valvular heart disease (54; 11.54%). The incidence of regional wall motion abnormalities showed no significant relation to age (p-value = 0.81). However, valvular heart disease significantly increased with age, affecting 14 (6.8%) of the 35-54 age group, 12 (11%) of the 55-64 age group, and 28 (20.3%) of those over 65 years (p-value = 0.001). Diastolic heart failure also showed a marked age-related increase, with the over-65 age group being the most affected (p-value = 0.001). Systolic heart failure was also more prevalent in older age groups, occurring in 17 (12.3%) of those over 65 years (p-value = 0.046).

**Table 2:** Echocardiographic findings categorized by age groups (n = 468) The p-value was calculated by chi-square.

Variables	Age groups					p-value	Test statistics
Echo findings	20-34 years	35-54 years	55-64 years	>65 years	Total		
Regional wall motion abnormalities							
Yes	0 (0%)	11 (5.4%)	6 (5.5%)	8 (5.8%)	25 (5.34%)	0.81	0.97
No	16 (100%)	194 (94.6%)	103 (94.5%)	130 (94.2%)	443 (94.66%)		
Valvular heart disease							
Yes	0 (0%)	14 (6.8%)	12 (11.0%)	28 (20.3%)	54 (11.54%)	0.0007	16.93
No	16 (100%)	191 (93.2%)	97 (89.0%)	110 (79.7%)	414 (88.46%)		
Diastolic heart failure							
Yes	0 (0%)	14 (6.8%)	19 (17.4%)	32 (23.2%)	65 (13.89%)	<0.0001	22.25
No	16 (100%)	191 (93.2%)	90 (82.6%)	106 (76.8%)	403 (86.11%)		
Systolic heart failure							
Yes	0 (0%)	10 (4.9%)	8 (7.3%)	17 (12.3%)	35 (7.48%)	0.046	7.97
No	16 (100%)	195 (95.1%)	101 (92.7%)	121 (87.7%)	433 (92.52%)		

Type of operations and correlation with clinicodemographic profile

Table 3 provides a summary of the association between clinicodemographic profile and types of surgery. The study showed that sex distribution demonstrated notable differences, with 70 (42.17%) of gastrointestinal tract (GIT) operations being male and 96 (57.83%) female. Age distribution also differed, with a relatively higher percentage of individuals aged >65 undergoing orthopedic operations (29; 35.73%). Furthermore, the presence of chronic diseases such as diabetes mellitus, hypertension, prior ischemic heart disease, and family history displayed varying prevalence rates across surgical interventions.

**Table 3:** Association between clinicodemographic characteristics and type of surgery (n = 468) The p-value was calculated by chi-square. GIT, gastrointestinal tract

Variables	GIT operations	Cardiac operations	Obstetric and gynecology	Orthopedic operations	Urinary system	Others	p-value	Test statistics
Gender								
Male	70 (42.17%)	43 (50.59%)	0 (0%)	39 (47.56%)	28 (57.14%)	19 (38%)	<0.0001	34.46
Female	96 (57.83%)	42 (49.41%)	36 (100%)	43 (52.44%)	21 (42.86%)	31 (62%)		
Age groups								
20-34	4 (2.41%)	4 (4.71%)	1 (2.78%)	3 (3.66%)	2 (4.08%)	2 (4%)	0.288	17.53
35-54	69 (41.57%)	36 (42.35%)	23 (63.89%)	35 (42.68%)	17 (34.69%)	25 (5%)		
55-64	37 (22.29%)	25 (29.41%)	6 (16.67%)	15 (18.29%)	17 (34.69%)	9 (18%)		
>65	56 (33.73%)	20 (23.53%)	6 (16.67%)	29 (35.37%)	13 (26.53%)	14 (28%)		
Residency								
Akre	14 (8.43%)	9 (10.59%)	2 (5.56%)	9 (10.98%)	5 (10.20%)	6 (12%)	0.594	17.91
Amedi	13 (7.83%)	9 (10.59%)	1 (2.78%)	5 (6.10%)	2 (4.08%)	4 (8%)		
Duhok	134 (80.72%)	63 (74.12%)	32 (88.89%)	63 (76.83%)	37 (75.51%)	37 (74%)		
Mosul	0 (0%)	2 (2.35%)	0 (0%)	1 (1.22%)	0 (0%)	0 (0%)		
Zakho	5 (3.01%)	2 (2.35%)	1 (2.78%)	4 (4.88%)	5 (10.20%)	3 (6%)		
Diabetes mellitus								
Yes	42 (25.30%)	17 (20%)	7 (19.44%)	21 (25.61%)	17 (34.69%)	20 (40%)	0.098	9.29
No	124 (74.70%)	68 (80%)	29 (80.56%)	61 (74.39%)	32 (65.31%)	30 (60%)		
Hypertension								
Yes	36 (42.40%)	84 (50.60%)	18 (50%)	37 (45.10%)	24 (48.00%)	27 (55.1%)	0.725	2.84
No	49 (57.60%)	82 (49.40%)	18 (50%)	45 (54.90%)	26 (52.00%)	22 (44.9%)		
Ischemic heart disease								
Yes	39 (23.49%)	12 (14.12%)	2 (5.56%)	18 (21.95%)	12 (24.49%)	13 (26.00%)	0.091	9.48
No	127 (76.51%)	73 (85.88%)	34 (94.44%)	64 (78.05%)	37 (75.51%)	37 (74%)		
Family history of cardiovascular disease								
Yes	13 (7.83%)	6 (7.06%)	1 (2.78%)	9 (10.98%)	6 (12.24%)	8 (16%)	0.283	6.24
No	153 (92.17%)	79 (92.94%)	35 (97.22%)	73 (89.02%)	43 (87.76%)	42 (84%)		

Correlation between echocardiographic findings and types of surgical procedures

Table 4 shows that across various surgical categories, distinct patterns emerged regarding the prevalence of echocardiographic findings. Regional wall motion abnormalities exhibited the highest in genitourinary system operations (4; 8.16%). Valvular heart disease also showed variability, being most common in orthopedic operations (13; 15.85%). The incidence of diastolic heart failure was relatively consistent, with the highest percentage observed in cardiac operations (12; 12.12%) and the lowest in GIT operations (30; 9.83%). Similarly, systolic heart failure was more prevalent in urinary system operations (6; 2.24%).

**Table 4:** Relationship between preoperative echocardiogram findings and types of surgical procedures (n = 468) The p-value was calculated by chi-square. GIT, gastrointestinal tract

Variables	GIT operations	Cardiac operation	Obstetric and Gynecology	Orthopedic operations	Urinary system operations	Others	p-value	Test statistics
Regional wall motion abnormalities								
Yes	9 (5.83%)	3 (3.53%)	1 (2.78%)	5 (6.10%)	4 (8.16%)	3 (6%)	0.859	1.93
No	157 (94.17%)	82 (96.47%)	35 (97.22%)	77 (93.90%)	45 (91.84%)	47 (94%)		
Valvular heart disease								
Yes	21 (13.83%)	6 (6.06%)	4 (11.11%)	13 (15.85%)	6 (12.24%)	4 (8%)	0.548	4.01
No	145 (86.17%)	79 (93.94%)	32 (88.89%)	69 (84.15%)	43 (87.76%)	46 (92%)		
Diastolic heart failure								
Yes	30 (9.83%)	12 (12.12%)	4 (11.11%)	9 (10.98%)	5 (10.20%)	5 (10%)	0.489	4.44
No	136 (81.17%)	73 (87.88%)	32 (88.89%)	73 (89.02%)	44 (89.80%)	45 (90%)		
Systolic heart failure								
Yes	13 (7.83%)	4 (4.71%)	1 (2.78%)	7 (8.54%)	6 (12.24%)	4 (8%)	0.566	3.89
No	153 (92.17%)	81 (95.29%)	35 (97.22%)	75 (91.46%)	43 (87.76%)	46 (92%)		

## Discussion

The study presents a comprehensive preoperative echocardiographic analysis of 468 patients, revealing significant insights into the population characteristics and the prevalence of various cardiac conditions among patients undergoing elective surgery in Duhok, Iraq. The study’s primary findings are as follows: first, clusters of cardiovascular risk factors were observed among surgical cases. Second, cardiac abnormalities were more prevalent among older surgical patients. Third, referrals for preoperative echocardiography were not solely driven by guideline-directed indications. Last, unremarkable findings on preoperative echocardiography were not uncommon.

The study highlights the varying prevalence rates of chronic conditions such as diabetes mellitus, prior ischemic heart disease, hypertension, and positive family history across different surgical cases. These chronic conditions are known to impact surgical outcomes and perioperative management. Diabetes mellitus was present in 124 (26.50%) of the study patients; this is in line with global trends of rising diabetes rates, which are known to significantly contribute to cardiovascular disease burden [12-15]. Hypertension, in particular, becomes more prevalent with advancing age, contributing to the development and progression of heart disease, including hypertensive heart disease and heart failure. Similarly, age-related changes in myocardial structure and function, compounded by the cumulative effects of cardiovascular risk factors, predispose individuals to systolic heart failure over time [16,17]. Moreover, about one-fifth of the study sample had a history of ischemic heart disease; this is in line with other studies conducted in Duhok, Iraq [18].

In our study, we observed a higher prevalence of echocardiographic abnormalities in middle-aged and elderly populations. This age distribution aligns with other studies, which have reported increased cardiovascular risk and prevalence of echocardiographic abnormalities in these age groups [19]. Furthermore, the study revealed notable associations between age and specific echocardiographic findings. For instance, regional wall motion abnormalities, valvular heart disease, hypertensive heart disease, and systolic heart failure were significantly increasing as the age of patients increased. These findings are consistent with previous studies that have demonstrated an age-related increase in these conditions due to the cumulative effects of risk factors and the natural aging process of the cardiovascular system [20-25].

The study highlights notable differences in sex distribution across different surgical categories, with a higher percentage of females undergoing gastrointestinal operations compared to males. This sex disparity in surgical utilization has been reported in previous studies and may reflect differences in disease prevalence, healthcare-seeking behavior, or provider referral patterns between sexes [26]. Understanding these sex-specific patterns is essential for tailoring surgical care and optimizing outcomes for both male and female patients.

Moreover, the study identifies variations in age distribution among patients undergoing orthopedic operations, with a relatively higher percentage of individuals aged over 65 years. This finding is consistent with the known association between aging and the prevalence of orthopedic conditions, such as osteoarthritis and fractures, which are more common in older adults [27]. The higher proportion of elderly patients undergoing orthopedic surgeries underscores the importance of considering age-related factors in perioperative management and optimizing care for this vulnerable population.

The highest incidence of systolic heart failure is observed in gynecologic and obstetric surgeries, whereas orthopedic operations exhibit the lowest prevalence. Similarly, the study highlights the variability in the prevalence of hypertensive heart disease and diastolic impairment across different surgical categories. Cardiac operations show the highest prevalence of hypertensive heart disease, while orthopedic operations exhibit the lowest. These findings are consistent with similar studies that have explored the associations between preoperative echocardiographic results and types of surgical procedures. For example, a study by Bright et al. observed similar patterns of variability in the prevalence of cardiac abnormalities across gynecologic and obstetric surgeries and different surgical categories [28].

Referrals for preoperative echocardiography in our region do not consistently adhere to guideline-directed indications. A notable proportion of cases were referred without clear clinical necessity, resulting in unremarkable findings. This trend may be influenced by medicolegal pressures and societal norms impacting healthcare practitioners, including surgeons and anesthesiologists.

Limitations

Our study has several limitations. First, it was a single-center study. Therefore, the results cannot be generalizable to broader populations due to regional variations in demographics, healthcare practices, and disease prevalence. Second, the cross-sectional design limits the ability to establish causal relationships between echocardiographic findings, chronic illnesses, and surgical outcomes. Third, the exclusion of patients with contraindications to echocardiography or implant devices may omit certain cardiac conditions, affecting the findings’ generalizability. Additionally, the study focuses on specific echocardiographic findings such as left ventricular systolic dysfunction, diastolic dysfunction, significant valvular abnormalities, and regional wall motion abnormalities, potentially overlooking other relevant cardiac conditions.

Recommendations

To improve cardiac outcomes in elective surgeries, it is essential to implement targeted cardiovascular screening for older adults to facilitate the early identification and management of age-related conditions. Strengthening the management of chronic diseases, such as diabetes, hypertension, and prior ischemic heart disease, is also critical prior to surgeries. Additionally, developing gender-specific health interventions and educational programs can help address the higher utilization of echocardiographic services among women. Enhancing healthcare access in districts outside Duhok will ensure equitable cardiac care, while bolstering primary care and referral systems will facilitate timely echocardiographic evaluations in tertiary hospitals throughout the region.

## Conclusions

This study highlights that echocardiographic abnormalities are significantly more prevalent in older patients undergoing elective surgeries, particularly in those aged 65 and above. Despite the value of echocardiography in preoperative assessment, many referrals lacked clear clinical indications, leading to a high rate of unremarkable findings. The study underscores the need for more judicious use of preoperative echocardiography, with a focus on targeted screening for high-risk patients, especially older adults, to optimize surgical outcomes and resource utilization.
